# Virtual reduction and 3D printing in the management of edentulous atrophic mandibular body fractures: case series and literature review

**DOI:** 10.1007/s10006-025-01484-7

**Published:** 2025-10-27

**Authors:** Ylenia Gugliotta, Fabio Roccia, Andrea Novaresio, Pompeo Cassano, Federica Sobrero, Guglielmo Ramieri, Emanuele Zavattero

**Affiliations:** 1 Department of Surgical Sciences, Maxillo - Facial Surgery Unit, AOU Città della Salute e della Scienza, University of Turin, Corso Bramante 8, Turin, Torino, 10126 Italy; 2 Department of Management and Production Engineering, Polytechnic University of Torino, Torino, Italy

**Keywords:** Mandibular fractures, Cad cam, Computer aided design, Edentulous mandible fracture, Atrophy

## Abstract

**Purpose:**

The management of atrophic mandibular fractures presents challenges due to the absence of stable occlusion and the insufficient bone volume. In this case series, we present a technique based on virtual surgical planning and in-house computer-aided design and manufacturing (CAD/CAM) for the surgical treatment of severe atrophic mandibular fractures.

**Methods:**

etween 1 January 2022 and 31 December 2024, eight patients with edentulous mandibular fractures were treated. Data collected included age, gender, cause of mandibular fracture, degree of atrophy according to Luhr's classification, site and type of fracture according to Ellis and Price, mean virtual planning and operative time, number of osteosynthesis plates, length of hospital stay, adequacy of reduction according to Ramanathan, and clinical outcomes.

**Results:**

Seven female and one male patient (mean age 82.6 ± 3.99 years; range 80-92) were included. Six patients were classified as having class III atrophy, and two as class II. Mild, moderate, and severe displacement were observed in three (37.5%), two (25%) and three (37.5%) patients respectively. Postoperative panoramic radiography demonstrated good and very good reduction in three (37.5%) and five (62.5%) patients respectively. Virtual surgical planning and plate pre-bending required an average of 90.5± 10.5 minutes. The mean operative time was 135.3 ± 6.63 minutes.

**Conclusion:**

Virtual fracture reduction and plate pre-bending on stereolithographic models represent a valuable tool for managing severe atrophic mandibular fractures. The authors believe this approach has potential to improve operative accuracy and reduce surgical time in these complex cases, offering good cost-effectiveness and time efficiency.

## Introduction

The increase in life expectancy across Europe and developing nations, together with the adoption of more active lifestyles and the consequent rise in injuries among the elderly, is leading to a rise in the incidence of maxillofacial fractures in patients over 60 years of age [1, 2]. This trend, combined with the growing prevalence of age-related conditions such as edentulism and mandibular atrophy, is contributing to a rise in the incidence of atrophic mandibular fractures [3, 4].

According to AO principles and the literature, the recommended treatment for severe atrophic mandibular fractures is open reduction and internal rigid fixation with a load-bearing plate and locking screws [4, 5].

This technique necessitates extensive exposure to achieve full visualisation of the bone fragments and allows the placement and adaptation of a long reconstruction plate and its fixation on adequate bone. Therefore, an extraoral approach is often recommended, leading to prolonged surgery time in elderly patients who often present with medical comorbidities [3–6].

Since the 1980 s, the application of computer-aided design and manufacturing (CAD/CAM) in healthcare has revolutionised diagnostic and interventional medicine [8]. Virtual surgical planning (VSP) initially found application in maxillofacial reconstructive and orthognathic surgery, and its advantages in efficiency and accuracy, as well as morphological and functional outcomes, are well documented in the literature [7–13].

In the past decade, several authors have highlighted the potential of these technologies in cranio-maxillofacial trauma surgery, particularly for reducing surgical time, guiding fracture reduction, and predicting reconstruction outcomes [14–17]. Notably, midface and orbital trauma have benefitted the most from these advancements, due to the complex surgical access required, the proximity to vital structures, and the high demands for both functional and aesthetic outcomes related to the eye [18].

Few authors have proposed the use of VSP and CAD/CAM in the treatment of mandibular fractures. The design of bone fragment repositioning guides [19] or occlusal splints based on an ideal occlusion achieved after virtual fracture reduction [20, 21] has been reported in the literature, but both these techniques rely on dental occlusion, and therefore are not applicable to edentulous patients.

The technique of virtual reduction for mandibular fractures has already been described in the literature [22], and recent reports have utilised virtual repositioning techniques and CAD/CAM technologies for the treatment of condylar fractures [23–25]. However, the management of edentulous atrophic mandibular fractures using virtual surgical planning (VSP) applications has been documented only in a few case reports [26, 27] or small case series [28–31].

The primary objective of this study was to present the outcomes of VSP combined with CAD/CAM and 3D printing techniques in the surgical management of eight cases of atrophic mandibular fractures. Additionally, a review of the current literature is presented to contextualize these findings and highlight advancements in this evolving field.

## Materials and methods

Patients with edentulous atrophic mandibular body fractures treated at the Division of Maxillofacial Surgery, AOU Città della Salute e della Scienza, University of Turin, Italy between 1 January 2022, 31 December 2024 were included in this study. Patients with pathological fractures, as defined by Ezsias and Sugar [32], or who had been treated with bisphosphonates or other drugs that might have had an adverse effect on bone remodelling were excluded.

Collected data included age, gender, cause of mandibular fracture, degree of atrophy according to Luhr’s classification [33], site and type of the fracture (non-displaced; mild, moderate, or severe displacement according to Ellis and Price [3] — the most displaced fracture per patient was considered), number of osteosynthesis plates, mean virtual planning time, 3D printing time, operative time, length of hospital stay, adequacy of reduction according to Ramanathan et al. [20], and complications. The minimum follow-up time was three months.

Descriptive statistical analysis was conducted using Microsoft Excel (Microsoft Corporation, Redmond, WA, USA). The software was used to calculate means, standard deviations, and ranges, where appropriate, in order to summarise patient demographics, fracture characteristics, as well as planning time, operative duration, and length of hospital stay. The chi-square test was performed using the same software application with the significance level set at 5% (α = 0.05).

### Pre-operative VSP

**Fig. 1:**
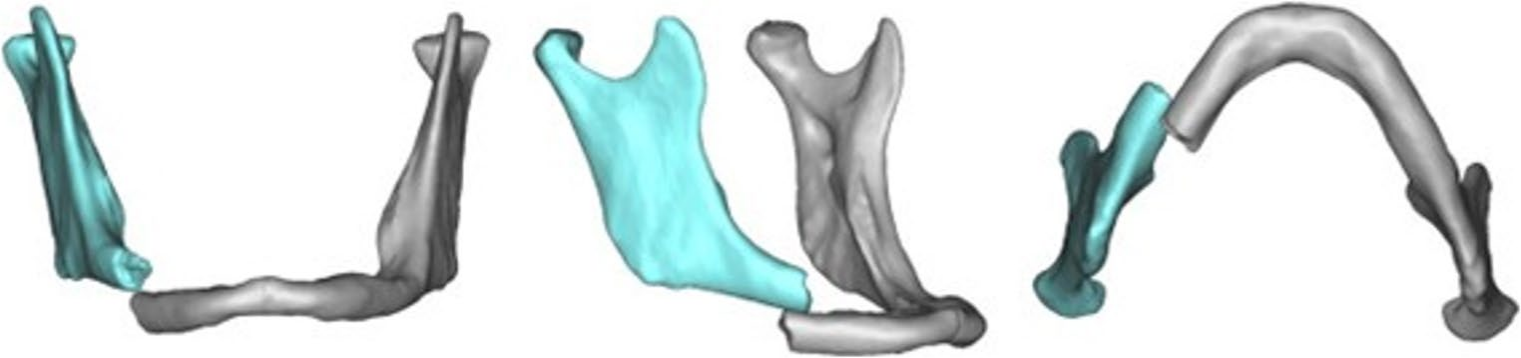
Patient no. 1. Atrophic mandible fracture segmented into individual fracture segments. This patient presented a quadruple mandibular fracture; three fractures were non-displaced and therefore were segmented as a single fracture segment

VSP was carried out as follows:

- Virtual model: DICOM (Digital Imaging and Communications in Medicine) data of the preoperative CT scan of each patient were transferred to Mimics Innovation Suite^®^ 24.0 (Materialise, Belgium) segmentation software. The thresholding and segmentation of the skull were performed. The mandibular bone was further segmented into individual fracture segments and their 3D stereolithography files (STL) were extracted. (Fig. 1).

**Fig. 2:**
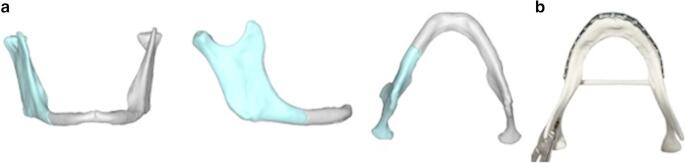
Patient no. 1. **a**. Anatomical model of the mandibular after virtual fracture reduction. **b**. Resulting mandibular model printed using a formlabs^®^ resin 3D printer and used to preoperatively pre-bend a stock load-bearing plate

Then, virtual reduction was carried out by manipulating the fracture segments in order to achieve optimal anatomical alignment using ProPlan CMFTM software (Materialise, Belgium) and an anatomical mandibular STL model was obtained. (Fig. 2a).

- 3D manufacturing: to transfer the plan information to the operating theatre the obtained anatomic mandible STL model was printed in our in-hospital 3D laboratory using FormLabs^®^ resin 3D printer. (Fig. 2b).
- Model surgery: A stock load-bearing device (MODUS^®^ TRILOCK, Medartis, Switzerland) was preoperatively pre-bent on the obtained stereolithographic model. (Fig. 2b).

### Surgical technique

**Fig. 3:**
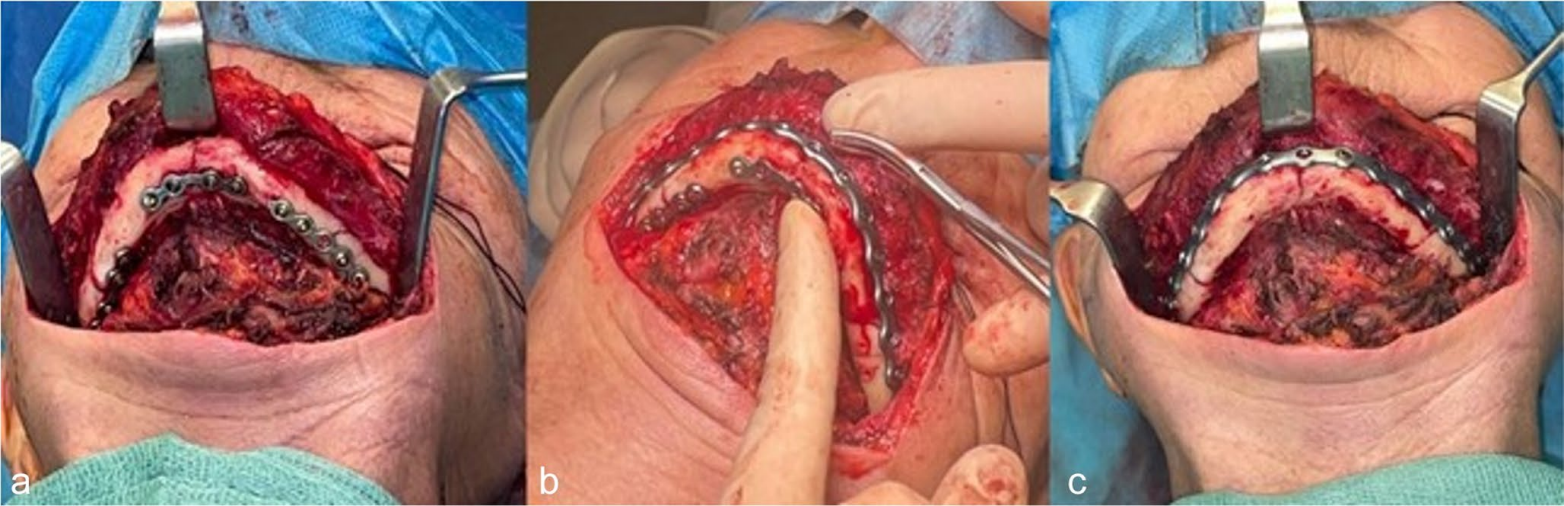
Patient n°1. Open reduction and internal fixation (ORIF) via a cervical approach. (**a**) fracture simplification and temporary fixation with 1.0 mm-thick miniplate with monocortical screws. (**b**) pre-bent 2.0 mm-thick angle to angle plate placed and fixed with locking screws. (**c**) final result following removal of the miniplates

All surgeries were performed by the same senior surgeon, with assistance from two residents. Open reduction and internal fixation (ORIF) using an external (submandibular) approach was performed. Dissection was carried out in a subperiosteal plane to expose the mandibular fracture site, with careful preservation of the marginal mandibular branch of the facial nerve. Preservation of the superior and lingual attachments of the periosteum was ensured to maintain the vascular supply. Each fracture reduction was achieved through temporary fixation using one 1.0 mm thick miniplate with monocortical screws (MODUS^®^ TRAUMA 2.0, Medartis, Switzerland), positioned along the inferior border of the mandible, to maximize bone surface contact (Fig. 3a). After reduction of all the fractures the pre-bent 2.0 mm thick plate was placed from the symphysis to the mandibular angle with at least three locking screws on each side of each fracture. In cases of bilateral fractures, an angle-to-angle plate was placed. (Fig. 3b). Then, miniplates were removed. (Fig. 3c).

The adequacy of reduction was evaluated intra-operatively as the degree of separation between the fracture fragments after osteosynthesis according to Ramanathan et al. [20].

**Fig. 4:**
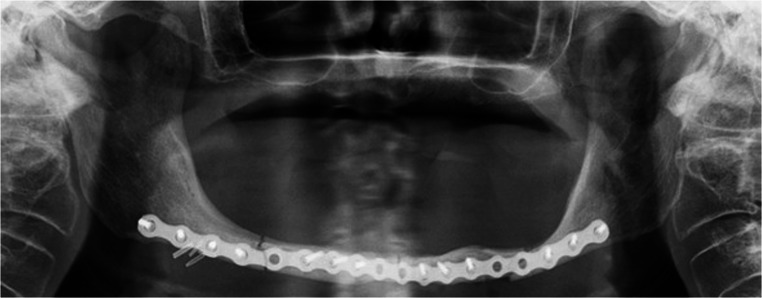
Patient n°1. Postoperative panoramic radiograph showing good reduction of the mandibular fractures

All patients underwent a postoperative panoramic radiograph and clinical examinations at one week, one month and at three months after surgery to assess the clinical outcomes. (Fig. 4).

##  Results

 Eight patients were included, seven (87.5%) were female. The mean age was 82.6 ± 3.99 years (range 80–92). The aetiology of trauma was a fall caused by slipping or tripping in all cases. Six patients were classified as having class III atrophy, and two as class II.

 All patients had multiple mandibular fractures, for a total of 19 fractures. The most common fracture site was the mandibular body (78.9%), which was involved in at least one fracture in every patient. Mild displacement was observed in three patients (37.5%), moderate displacement in two (25%), and severe in three (37.5%). Virtual surgical planning and plate pre-bending took on average 90.5± 10.5 minutes, but the time decreased from the first to the last patient (Table 1). Mean resin 3D printing time was 208 ± 3.4 minutes.

**Table 1:** Characteristics of patients and fractures presented

*N*.	Gender	Age (y)	*N*° of F.	F. site	F. Side	Atrophy degree^a^	Displacement^b^	Adequacy of reduction^c^	Planning time (m)
1	F	83	4	Body, Symphysis	Bilateral	III	Mild	Good	110
2	F	80	2	Body	Bilateral	III	Severe	Very good	106
3	F	83	2	Body, Condyle	Right	II	Mild	Good	90
4	F	92	2	Body	Bilateral	III	Severe	Very good	86
5	F	80	2	Body, Parasymphysis	Bilateral	II	Moderate	Very good	85
6	M	80	2	Body	Bilateral	III	Moderate	Very good	85
7	F	82	3	Body	Bilateral	III	Severe	Very good	82
8	F	81	2	Body	Bilateral	III	Mild	Good	80

*F* fracture, *y* years, *m* minutes

^*a*^ According to Luhr, 1996 [33]

^b^ According to Ellis and Price, 2008 [3]

^c^ According to Ramanathan et al., 2020 [20]

 Intraoperatively, the pre-bent plate demonstrated a good anatomical fit in all patients following proper reduction, with no need for further plate adaptation. The adequacy of reduction was judged intraoperatively as“very good” in five patients (62.5%) and “good” in three (37.5%) and was significantly correlated with the extent of displacement (p=0.02).

 The mean operative time was 135.3 ± 6.63 minutes. Postoperative panoramic radiographs showed adequate reduction in all patients. The average post-surgery hospital stay was 2 ± 0.76 days, contributing to an overall hospitalization duration of 4.6 ± 1.77 days. All patients experienced postoperative oedema and pain, which resolved within one week after surgery. None exhibited delayed wound healing, wound dehiscence, seroma, haematoma, or infection. At three months post-surgery, no patients exhibited injury to the marginal mandibular branch of the facial nerve, to the mandibular nerve or hypoesthesia of the lower lip or chin . The mean follow-up duration was 16 months.

##  Discussion

 Edentulous mandibular fractures account for less than 5% of all mandibular fractures; however, they present unique clinical challenges [34]. The progressive alveolar bone resorption associated with edentulism results in reduced and sclerotic bone volume, poor vascularisation, and the absence of stable anatomical landmarks [35]. Additionally, the lack of occlusal support eliminates a critical reference point for proper alignment of fracture segments and restoration of vertical dimension and the atrophic bone structure complicates the placement of rigid reconstruction plates and increases the risk of injury to the inferior alveolar nerve (IAN). These anatomical and biomechanical limitations make accurate anatomical reduction more difficult and raise the risk of local complications.

 Moreover, edentulous patients with mandibular atrophy are often elderly and frequently present with multiple comorbidities, which heighten both surgical and anaesthetic risks [23, 29–31]. In light of these complexities, the use of virtual surgical planning (VSP) and computer-aided design/computer-aided manufacturing (CAD/CAM) technologies has emerged as a promising strategy to enhance the management of atrophic mandibular fractures [36, 37].

 As early as 2010, Van Sickels and Cunningham reported favourable outcomes in mandibular fracture management using preoperatively bent plates on standard plastic mandibular models, which were subsequently adjusted intraoperatively to match patient-specific anatomy [38]. Although VSP was not employed in their study, the authors noted a reduction in operative time when plates were pre-contoured before surgery. In recent years, with the progressive development of new technologies, several authors have advocated the application of VSP and CAD/CAM-assisted approaches in the treatment of fractures of the atrophic edentulous mandible (Table 2) [22, 31].

**Table 2:** Literature review

	Patient (*N*, gender)	Fractures (*N*, site)	Atrophy degree ^a^	Surgical technique	Outcomes
Oliveira Brito et al., 2016 [27]	1, M	1, body	n.r.	EO approach, LBO	Satisfactory reduction^*b*^
Abbate et al., 2023 [28]	4, 50% F	n.r.	n.r.	EO approach, LBO	Accuracy: 100%^*c*^
Caruso et al., 2024 [31]	5, 66% F	2, body + symphysis	III	EO approach, LBO	Accuracy: 88.2%^*d*^
		1, body			
		3, bil body, symphysis			
		1, body			
		2, body + symphysis			
Façanha de Carvalho et al., 2020 [26]	1, F	2, bil body	III	EO approach, LBO	Good reduction^*e*^
Maloney et al., 2019 [30]	2, F	2, bil body	III	EO approach, LBO	Good reduction^*e*^
		1, body			Adequate reduction^*e*^
Castro-Núñez et al., 2018 [29]	2, 50% F	1, body	n.r.	EO approach, LBO + temporary miniplates	n.r.
		1, body			

*M* male, *F* female, *bil* bilateral, n.r.: not reported, *EO* extra-oral, *LBO* load bearing osteosynthesis

^*a*^ According to Luhr, 1996 [33]

^*b*^ Evaluated on postoperative CT

^*c*^ Reported as percentage of cases with discrepancies between predicted and postoperative bone segments position < 1.5 mm

^*d*^ Reported as percentage of cases with discrepancies between predicted and postoperative bone segments position < 2 mm

^*e*^ Evaluated on postoperative panoramic radiograph

 A review of the literature confirms that these fractures occur more frequently in females and are most commonly located in the mandibular body. In all reported cases, the fractures were approached via an extra-oral route and treated using load-bearing osteosynthesis. Virtual surgical planning (VSP) was consistently employed preoperatively to achieve virtual reduction of the fracture segments and to generate an STL model of the reconstructed mandible. This model was then 3D printed and used to pre-bend a load-bearing plate prior to surgery, with the exception of five cases reported by Caruso et al. [31].

 In this case series, the digital mandibular model was utilised to design patient-specific titanium plates and drilling guides with notably high precision, achieving fragment positioning errors of less than 2 mm in 88.2% of cases. The authors also reported that the use of patient-specific implants (PSIs) facilitated accurate condylar seating and provided high predictability in screw placement, thereby reducing the risk of injury to the inferior alveolar nerve (IAN). Moreover, these custom plates were described as thinner than standard stock plates while maintaining sufficient mechanical strength, thus minimising interference with future dental rehabilitation. The authors emphasised that this technique is particularly beneficial in cases of severe atrophy, comminuted fractures, or when bone grafting is required to bridge fracture gaps, as observed in several cases within the series [31]. However, the production of titanium PSIs remains both costly and time-consuming. Additionally, the design of accurate and unambiguous drilling guides can be particularly challenging in the absence of dental reference points.

 On the other hand, Façanha de Carvalho et al described the use of a pre-bent plate together with fragments repositioning guides [22]. However, in cases of non-comminuted fractures and without bony gaps, we believe that the fracture can be anatomically reduced using the pre-bent plate as a reference for proper positioning. Moreover, the repositioning guide described does not incorporate drilling references, therefore its primary advantage is stabilising the fracture prior to placing the load-bearing plate; this can be achieved more efficiently and cost-effectively with the use of miniplates. In our study the use of a stock plate bent preoperatively on mandible model obtained from the virtual anatomic reduction of the fractures allowed for good reduction in all cases. Moreover, the in-house printing of the models enabled maintaining low costs, and the planning times were relatively short, did not depend on the number of fractures and decreased with the learning curve and training of dedicated personnel. Additionally, this process eliminated the lengthy intraoperative bending of thick plates. Although this study does not include an accuracy analysis, the same protocol demonstrated a very high accuracy in the case series by Abbate et al., where all the cases showed discrepancies of less than 1.5 mm between the virtual planning and postoperative CT [28].

 On the other hand, in cases of comminuted fractures or those requiring bone grafts, when anatomic reduction is impossible, the use of drilling guides and custom plates as described by Caruso et al [31] could offer greater advantages, justifying the higher costs and production times of the PSIs.

 The integration of new technologies in the management of atrophic mandibular fractures offers a tailored approach that addresses many challenges associated with these complex injuries, with a potential reduction in operative times in a vulnerable population and a potential improvement in the clinical outcomes.

 The progressive availability of specific in-house tools for virtual surgical planning and 3D printing has allowed a simpler, quicker, and more cost-effective utilisation of these technologies. Furthermore, with a short learning curve, the in-hospital 3D laboratory could also be considered a valuable tool for learning, as the virtual model allows the junior surgeons to simulate the operation and share the decision making with the senior surgeons. The implementation of preoperative planning enhances the accessibility of the procedure for less experienced surgeons, enabling them to perform it safely and achieve favourable outcomes even in their early cases. This is especially advantageous given the condition's low epidemiological prevalence, which limits the opportunities for younger surgeons to gain practical experience [4, 22, 29, 30].

 Despite the numerous advantages, the higher costs compared to conventional techniques are a significant consideration. Furthermore, the planning and fabrication process requires preparation time that can delay surgery by several days or weeks. However, for atrophic edentulous fractures, there is rarely an indication for immediate emergency treatment, making a brief delay usually not clinically significant.

 Despite the limited number of patients, this case series represents the largest cohort of edentulous atrophic multiple mandibular fracture patients treated with the aid of VSP.

 Beyond the already acknowledged limitation of the small sample size, this study presents additional constraints. Firstly, its retrospective nature inherently limits the strength of the conclusions that can be drawn. Secondly, in the absence of postoperative CT scans, the assessment of fracture reduction adequacy relied solely on the intraoperative judgement of the operating surgeon. This introduces a potential source of subjectivity and variability, which could be addressed in future studies through more objective accuracy assessments. Prospective, and ideally multicentre, investigations would allow for a larger and more representative cohort, particularly given the relatively low incidence of this specific pathology. In such studies, the inclusion of postoperative CT imaging would enable a more robust analysis of accuracy by allowing for a direct comparison between the 3D models of the mandible reconstructed after virtual fracture reduction and those obtained following osteosynthesis. The superimposition and comparison of these models could provide valuable insights into the actual precision of preoperative plate bending on stereolithographic replicas, and may further elucidate whether such an approach has a statistically significant impact on clinical outcomes—such as the potential reduction in the incidence of inferior alveolar nerve injuries when compared to cases treated without digital planning tools.

##  Conclusion

 In conclusion, we believe that the use of a plate preoperatively bent on a stereolithographic model of the patient's mandible, obtained through virtual reduction of the fractures, should become part of the standard treatment protocol for atrophic mandibular fractures— especially when in-house 3D printing is possible. In cases of comminuted fractures or with significant bone continuity deficits, the use of drilling guides and customised plates should be considered.

 It is important to consider that virtual surgical planning and the 3D printing workflow require a learning curve for digital planning and a 3D laboratory workstation with specialised staff, equipment, and software. Further prospective studies comparing VSP and conventional intraoperative plate bending are needed to assess differences in surgical time, costs, and clinical outcomes.

## Data Availability

The authors confirm that the data supporting the findingsof this study are available within the article. Any further data isavailable from the corresponding author, YG, upon reasonable request.

## Declarations

This is an observational study. The Intercompany Territorial Ethics Committee (CET), A.O.U. Città della Salute e della Scienza di Torino, Turin, Italy has confirmed that no ethical approval is required. Informed consent was obtained from all individual participants included in the study.

This study was performed in line with the principles of the Declaration of Helsinki.

The authors affirm that human research participants provided informed consent for publication of the images in Figure(s) 1, 2, 3 and 4.

The authors confirm that the data supporting the findings of this study are available within the article. Any further data is available from the corresponding author, YG, upon reasonable request.
